# Effect of femoral posterior condyle offset on knee joint function after total knee replacement: a network meta-analysis and a sequential retrospective cohort study

**DOI:** 10.1186/s13018-021-02233-8

**Published:** 2021-02-10

**Authors:** Yimin Zhang, Jun Wang, Miao Zhang, Yun Xu

**Affiliations:** Department of Orthopedic Surgery, Weifang People’s Hospital, No. 151, Guangwen Street, Weifang, 261000 Shandong P.R. China

**Keywords:** Total knee replacement, Posterior femoral condyle offset, Cohort study, Bayesian network model, Network meta-analysis

## Abstract

**Background:**

This study was conducted with the aim to compare the effect of posterior condyle offset (PCO) changes on knee joint function of patients following total knee replacement (TKR).

**Methods:**

Electronic and manual searches were performed in the PubMed, Embase, and Cochrane Library databases from inception to September 2019. Network meta-analysis combined direct and indirect evidence to assess the weighted mean difference (WMD) and surface under the cumulative ranking curves (SUCRA) of different PCO changes (PCO ≤ − 2 mm, − 2 mm < PCO < 0 mm, 0 mm ≤ PCO < 2 mm and PCO ≥ 2 mm) on knee joint function after TKR. Then 103 OA patients undergoing unilateral TKR were included and the effect of PCO on the postoperative knee function was examined.

**Results:**

Totally, 5 cohort studies meeting the inclusion criteria were enrolled in this analysis. The results of meta-analysis showed that patients with 0 mm ≤ PCO < 2 mm after TKR had a better recovery of joint function (flexion contracture: 28.67%; KS functional score: 78.67%; KS knee score: 75.00%) than the remaining three groups. However, the knee flexion (77.00%) of patients with PCO ≤ − 2 mm after TKR was superior to the other three groups. Retrospective study also revealed a significant correlation between PCO changes and the flexion contracture, further flexion and KS functional score of patients after TKR, in which each functional knee score of patients with 0 mm ≤ PCO < 2 mm was better than the others.

**Conclusion:**

These findings suggest a close correlation between PCO magnitude and knee joint function after TKR and that 0 mm ≤ PCO < 2 mm is superior to other changes for joint function after TKR.

**Supplementary Information:**

The online version contains supplementary material available at 10.1186/s13018-021-02233-8.

## Background

Osteoarthritis (OA) is a kind of joint degenerative disease and often results in serious pains and loss of joint function, mainly afflicting the middle-aged and elderly population [1, 2]. The disease has a complex and multifactorial epidemiology with genetic, biological, and biomechanical components [3]. The incidence and prevalence of OA are increasing possibly attributed to the age, sex, obesity, and genetics of the population as well as abnormal loading of joints [4]. Unfortunately, the prevalence will rise over the next several decades in a continual manner, seriously affecting social health and economic costs along with daily activities and life quality of patients [5]. Total knee replacement (TKR) is a widely used surgical approach for patients with OA [6, 7]. It has been shown to contribute to a greater improvement in the pains, knee function, and quality of life in patients with late-stage knee OA [8]. However, TKR fails to restore the full range of motion of the knee joint [9], that is to say, the motion range of the knee joint post TKR is an important factor to determine the postoperative function of patients [10].

Posterior condylar offset (PCO) has been highlighted as a critical consideration for surgeons before operation in selecting the design and size of femoral component due to its greatest effect on final range of knee movement following TKR [11, 12]. The changes of PCO have a correlation with postoperative changes in flexion angle in knees following cruciate-retaining TKR [13]. A 3-mm decrease of the PCO has been found to be capable of reducing knee flexion by 10° before the occurrence of tibiofemoral impingement [9]. In addition, excess femoral PCO (4.7-fold greater than that of healthy knees) in TKR prostheses probably leads to knee joint flexion contracture because of the relative shortened posterior soft tissues [14]. The postoperative increase of PCO has been shown to induce posterior cruciate ligament overstretching and poor flexion angle of the knee following cruciate-retaining TKR [15]. Moreover, in a total of 16 cases undergoing navigated TKR due to primary knee OA, seven of them with the PCO of more than 2 mm present with a midflexion instability [16]. By contrast, ± 1-, ± 2-, and ± 3-mm PCO models in the posterior direction cause a decrease in the maximum patellofemoral contact stress and quadriceps force, yet an increase in the collateral ligament force [17]. In view of the inconsistent data reported on the effect of the femoral PCO on the knee function after TKR, we thus performed a network meta-analysis and a sequential retrospective cohort study to compare the effect of PCO changes on knee joint function of patients following TKR.

## Materials and methods

### Literature search

Electronic searches were performed in the PubMed, Embase, and Cochrane Library databases from the inception to September 2019. We also searched for relevant studies that were missed in the initial electronic search by conducting a manual search of cross-references. The manual search was conducted using keywords combined with free words, mainly including TKR, PCO, total knee arthroplasty and cohort study, etc.

### Inclusion and exclusion criteria

The inclusion criteria were (1) study design: cohort study; (2) intervention measures: PCO ≤ − 2 mm (change of PCO decrease of 2 mm or more), − 2 mm < PCO < 0 mm (change of PCO decrease by 0 mm to 2 mm), 0 mm ≤ PCO < 2 mm (change of PCO increase of 0 mm to 2 mm), PCO ≥ 2 mm (change of PCO increase by 2 mm or more); (3) study subjects: patients with OA or rheumatoid arthritis, etc. and received corresponding operation; patients had primary TKA for knee OA classified into grade 3 and 4, according to Kellgren-Lawrence grading system [18]; and (4) end outcomes: flexion or Knee Society score. The exclusion criteria were (1) patients with affected knee underwent open surgery or had a history of fracture; (2) patients with less than 1 year of follow-up; (3) patients with severe osteoporosis; (4) literature with incomplete data (such as non-matched study); (5) non-cohort studies; (6) duplicated publications; (7) conference reports, system assessments or abstracts; and (8) non-English literature.

### Data extraction and quality evaluation

With uniform data collection sheets, two reviewers independently extracted information from the selected studies. Any disputes regarding the extraction of data were resolved by agreement among several investigators. Literature quality was assessed by over two reviewers in accordance with the Newcastle-Ottawa Scale (NOS) [19]. The total score was 9 points and studies with more than 5 points could be included in the meta-analysis.

### Patient data

We recruited 103 patients (39 males and 64 females, aged 49–74 years with a mean age of 65.01 ± 5.90 years) with OA who had undergone knee replacement at Weifang People’s Hospital from March 2016 to March 2019 into the study. The PCO of all patients before and after operation was recorded. According to the changes of PCO before and after operation, patients following TKR were divided into 4 groups: 25 cases with PCO ≤ − 2 mm, 43 cases with − 2 mm < PCO < 0 mm, 43 cases with 0 mm ≤ PCO < 2 mm, and 8 cases with PCO ≥ 2 mm. Patients in the four groups were followed-up for 12 months to compare the differences of clinical evaluation indexes, such as Western Ontario and McMaster Universities (WOMAC), American Knee Society (AKS), knee-flexion range, and flexion contracture. The inclusion criteria for selection of patients were as follows: (1) patients diagnosed with OA of the knee and received knee replacement; (2) the replacement prosthesis was the type of posterior stabilized; (3) flexion contracture ≤ 15°, and no knee inversion before operation; (4) body mass index (BMI) of 20–35 kg/m^2^; (5) normal muscle strength of quadriceps femoris; (6) the patella without any treatment (sometimes only the larger osteophyte was removed); and (7) no drainage tube was placed during the operation. Patients were excluded if they met any of the following criteria: (1) patients with severe osteoporosis; (2) a history of fracture or open surgery of knee joint; (3) obvious osteophytes in the joint after operation; (4) ligament dysfunction around knee joint; and (5) lost to follow-up or incomplete/missing intraoperative data. This study was approved by the ethics committee of Weifang People’s Hospital, and all patients signed the informed consents prior to the study.

### Operative methods

All operations were performed by the same experienced doctors. After routine disinfection and anesthesia, a pneumatic tourniquet was applied to femoral and an incision was made in the medial anterior knee from 1/3 of the inner quadriceps tendon to the inner edge of the tibial tubercle and entering the joint bypassing the inner edge of the patella. The osteophyte, the fat pad under the patella, and the meniscus were removed. The osteotomy was then performed using specialized instruments, soft tissues were appropriately loosened, and the prosthesis was placed. The posterior cruciate ligament was released properly according to its tightness. Next, the wound was sutured intradermally, and 0.5 g of tranexamic acid was injected into the joint, which was covered with sterile dressing. Afterwards, the wound was treated with pressure bandage and sutured. After the operation, the patients were given active anti-infection and other symptomatic treatments.

### Observation indexes

All patients were followed up for 12 months. At the 1st, 2nd, 3rd, 6th, and 12th month after operation, the outpatient reexamination was carried out. Standard lateral X-ray was photographed and PCO, flexion contracture, and flexion of knee joint were measured. At the same time, AKS and WOMAC scores were used to evaluate the knee joint and OA. The former included knee joint score and function score, each accounting for 100 points. The higher score reflected better evaluation. The lower score of the latter was an indicative of better knee function.

### Statistical analysis

We first conducted traditional pairwise meta-analyses for studies that directly compared different treatment arms. The pooled estimates of weighted mean difference (WMD) and 95% credible intervals (CrIs) were reported in our results. Chi-square test and *I*-square test were used to test heterogeneity among the studies. Second, we used the R Software (version 3.2.1) to plot the network diagram, in which each node represented various interventions, the node size represented the sample size, and the line thickness between nodes represented the number of included studies. Third, we performed Bayesian network meta-analyses to compare different interventions to each other. Each analysis was based on non-informative priors for effect sizes and precision. Convergence and lack of auto correlation were checked and confirmed after four chains and a 20,000-simulation burn-in phase. Finally, direct probability statements were derived from an additional 50,000-simulation phase. We used the node-splitting method to estimate consistency between the direct evidence and indirect evidence. Based on the results, a consistency or an inconsistency model was selected. When the results of node-splitting methods were *p* > 0.05, a consistency model was selected for the analysis. To assist in the interpretation of WMDs, we calculated the probability of each intervention being the most effective treatment method based on a Bayesian approach using probability values summarized as surface under the cumulative ranking curve (SUCRA). The larger the SUCRA value, the better the rank of the intervention**.** All computations were done using R (V.3.2.1) package gemtc (V.0.6), along with the Markov Chain Monte Carlo engine Open BUGS (V.3.4.0). All clinical data were processed using SPSS 21.0 statistical software (IBM Corp., Armonk, New York, USA). The measurement data were expressed as mean ± standard deviation. Data among multiple groups were compared by one-way analysis of variance (ANOVA), followed by Tukey’s post hoc tests with corrections for multiple comparisons. Enumeration data were analyzed by chi-square test. *p* < 0.05 indicated that the difference was statistically significant.

## Results

### Baseline characteristics of included studies

**Fig. 1:**
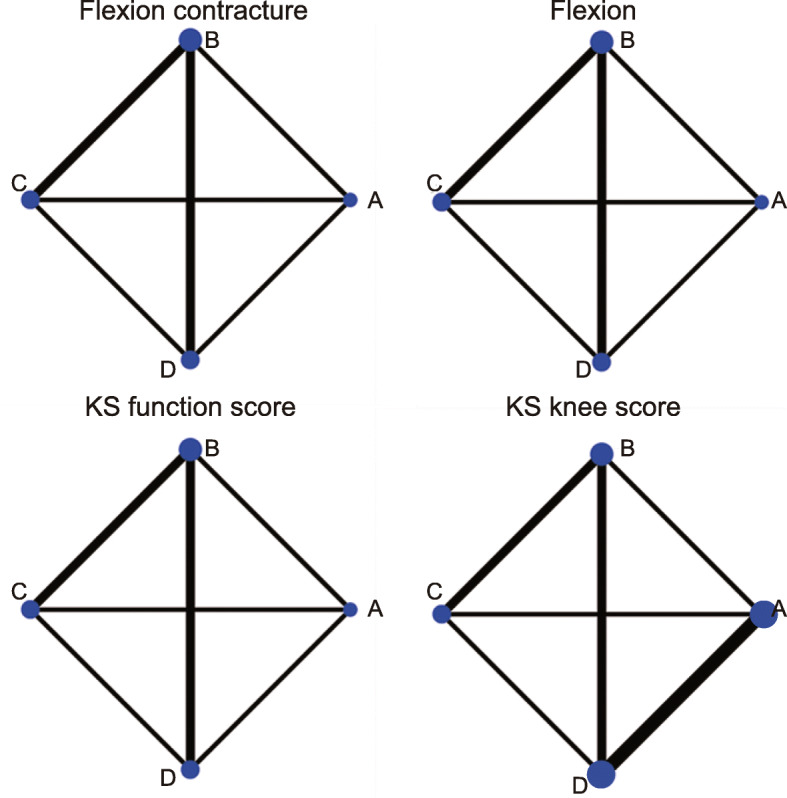
Relationship between PCO changes and knee joint function of patients after TKR. *KS* knee society. **a**, The change of PCO decrease of 2 mm or more. **b**, The change of PCO decrease by 0 mm to 2 mm. **c**, The change of PCO increase by 0 mm to 2 mm. **d**, The change of PCO increase by 2 mm or more

**Fig. 2:**
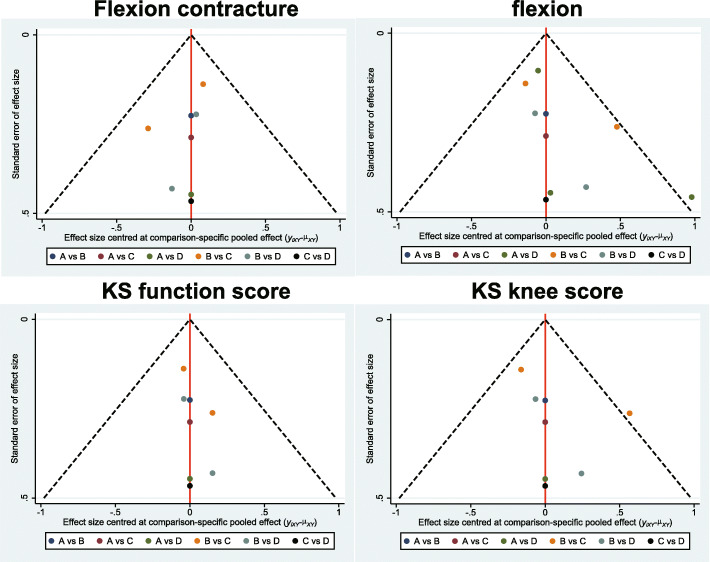
Bias analysis of effects of PCO changes on knee joint function of patients after TKR. *KS* knee society. **a**, The change of PCO decrease of 2 mm or more. **b**, The change of PCO decrease by 0 mm to 2 mm. **c**, The change of PCO increase by 0 mm to 2 mm. **d**, The change of PCO increase by 2 mm or more

Through electronic and manual searches, 989 articles were found. After the initial screening, we excluded 131 duplicated articles, 262 letters or summaries, 135 non-human studies, and 187 non-English articles. For the remaining 274 articles, after detailed assessment of the full text, we excluded 140 non-cohort studies, 126 articles that were irrelevant with TKR, and 3 articles with incomplete data. Eventually, 5 eligible cohort studies that were published between 2009 and 2018 were included for this network meta-analysis (Supplementary Figure 1). It included a total of 829 patients with OA or rheumatoid arthritis and undergoing corresponding operations. Among them, the number of patients with PCO ≥ 2 mm after TKR was relatively large (Fig. 1). The study subjects in the included 2 studies were from European and American populations, and those in 3 studies were from Asian populations. Among the 5 studies, 1 study was four-arm trial and 4 studies were two-arm trials. The baseline characteristics of included studies are shown in Table 1, and the NOS for literature quality assessment is displayed in Supplementary Figure 2. Bias analysis is shown in Fig. 2. The standard meta-analysis checklist is shown in Supplementary Figure 3 [25].

**Table 1:** The baseline characteristics of included studies

First author	Year	Country	Interventions				Total	Sample size					Age (years)			PMID	Type of study
			T1	T2	T3	T4		T1	T2	T3	T4	T1	T2	T3	T4		
Seo SS [20]	2009	Korea	A	B	C	D	111	31	54	20	6	66.2 (52–72)	66.2 (52–72)	66.2 (52–72)	66.2 (52–72)	19835308	Corhot study
Geijsen GJ [21]	2014	Germany	A	D	–	–	27	16	11	–	–	67 ± 9.6	67 ± 9.6	–	–	23677140	Corhot study
Wang JT [22]	2015	China	B	D	–	–	89	31	58	–	–	62.14 ± 5.37	61.07 ± 5.74	–	–	26777708	Corhot study
Degen RM [23]	2017	England	A	D	–	–	391	245	146	–	–	76 ± 9	76 ± 9	–	–	29183086	Corhot study
Lee OS [24]	2018	Korea	B	C	–	–	211	104	107	–	–	72.1 ± 7.1	71.9 ± 7	–	–	29195851	Corhot study

A, The change of PCO decrease of 2 mm or more. B, The change of PCO decrease by 0 mm to 2 mm. C, The change of PCO increase by 0 mm to 2 mm. D, The change of PCO increase by 2 mm or more. *RCT* randomized controlled trial

### Network meta-analysis reveals a better recovery of joint function in patients with 0 mm ≤ PCO < 2 mm after TKR

**Fig. 3:**
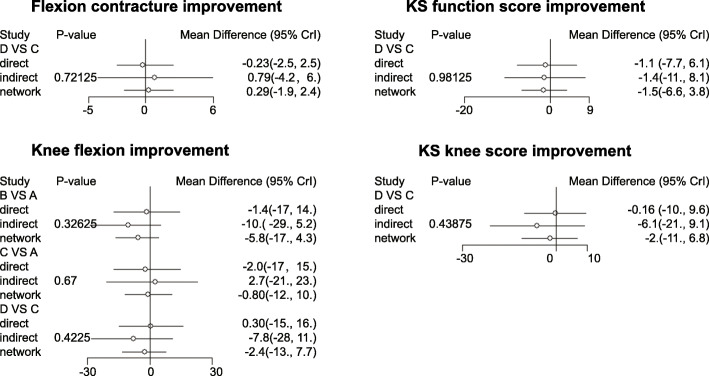
Node-splitting diagrams of the effects of PCO changes on knee joint function of patients after TKR. **a**, The change of PCO decrease of 2 mm or more. **b**, The change of PCO decrease by 0 mm to 2 mm. **c**, The change of PCO increase by 0 mm to 2 mm. **d**, The change of PCO increase by 2 mm or more

The consistency analysis for the four outcome indexes (flexion contracture, knee flexion, KS functional score, and KS knee score) by the node-splitting method showed that the results of direct and indirect evidence were consistent (all *p* > 0.05, Fig. 3), and therefore consistency model was selected for the subsequent analysis.

**Fig. 4:**
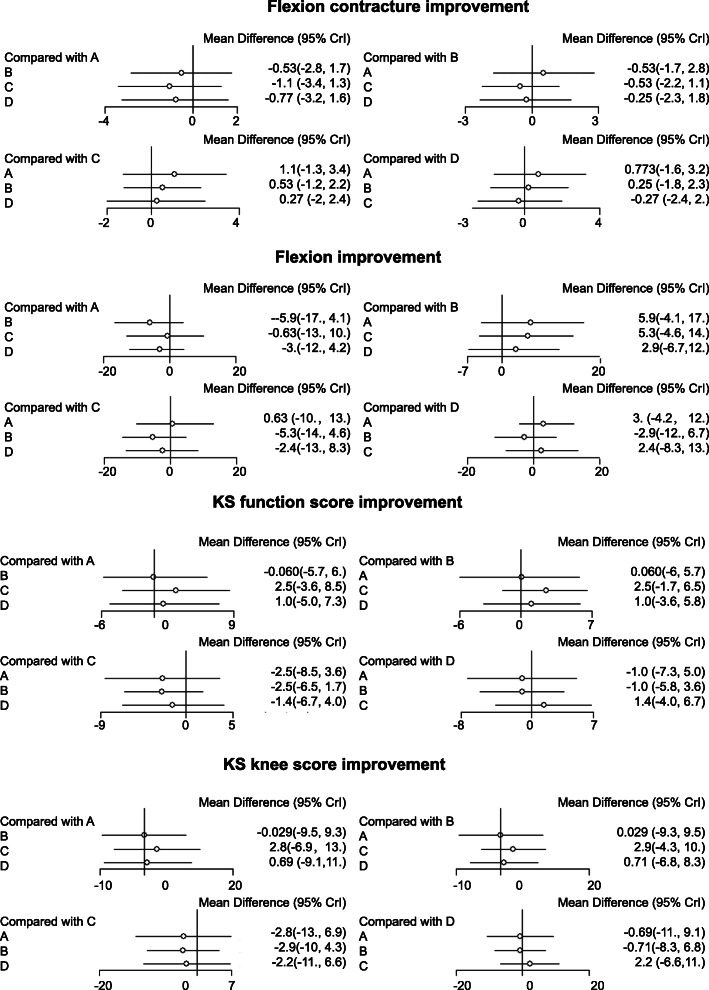
A forest map of the effects of PCO changes on knee joint function of patients after TKR. **a**, The change of PCO decrease of 2 mm or more. **b**, The change of PCO decrease by 0 mm to 2 mm. **c**, The change of PCO increase by 0 mm to 2 mm. **d**, The change of PCO increase by 2 mm or more

As shown in Table 2, the improvement value of knee flexion in patients with 0 mm ≤ PCO < 2 mm was relatively high (WMD = 6.00, 95% CI = 2.49–9.50), indicating that 0–2-mm increase of PCO was relatively good for the knee joint function after TKR. However, as shown in Fig. 4 and Table 3, there was no significant difference in the improvement values of flexion contracture, knee flexion, KS functional score, and KS knee score of patients following TKR in each group (all *p* > 0.05).

**Table 2:** Estimated WMD and 95% CI from pairwise meta-analysis in terms of flexion contracture improvement, flexion improvement, KS function score improvement, and KS knee score improvement

Included studies	Comparisons	Pairwise meta-analysis		
		WMD (95% CI)	I2	Ph
Flexion contracture improvement				
2 studies	C VS. B	− 0.49 (− 1.72− 0.75)	63.2%	0.10
2 studies	D VS. B	0.22 (− 1.40–1.85)	0%	0.63
Knee flexion improvement				
3 study	D VS. A	− 1.54 (− 3.97–0.88)	64.6%	0.06
2 studies	C VS. B	6.00 (2.49–9.50)	84.4%	0.01
2 studies	D VS. B	2.37 (− 1.83–6.58)	12.9%	0.28
KS function score improvement				
2 studies	C VS. B	2.36 (− 0.92–5.64)	0%	0.40
2 studies	D VS. B	0.51 (− 3.79–4.80)	0%	0.52
KS knee score improvement				
2 studies	C VS. B	2.37 (− 0.16–4.89)	91.80%	< 0.01
2 studies	D VS. B	− 1.16 (− 4.18–1.84)	0%	0.38

*WMD* weighted mean difference, *95*% *CI* 95% confidence intervals, *KS* knee society. A, The change of PCO decrease of 2 mm or more. B, The change of PCO decrease by 0 mm to 2 mm. C, The change of PCO increase by 0 mm to 2 mm. D, The change of PCO increase by 2 mm or more

**Table 3:** WMD and 95% confidence intervals of four treatment modalities of two endpoint outcomes

WMD (95% CI)			
Flexion contracture improvement			
A	− 0.54 (− 2.79, 1.58)	− 1.06 (− 3.41, 1.23)	− 0.79 (− 3.28, 1.54)
0.54 (− 1.58, 2.79)	B	− 0.48 (− 2.25, 1.16)	− 0.21 (− 2.39, 1.78)
1.06 (− 1.23, 3.41)	0.48 (− 1.16, 2.25)	C	0.33 (− 1.97, 2.43)
0.79 (− 1.54, 3.28)	0.21 (− 1.78, 2.39)	− 0.33 (− 2.43, 1.97)	D
Knee flexion improvement			
A	− 5.84 (− 16.67, 3.72)	− 0.66 (− 13.21, 9.90)	− 2.97(− 11.85, 4.02)
5.84 (− 3.72, 16.67)	B	5.28 (− 4.75, 14.38)	2.93 (− 6.41, 11.61)
0.66 (− 9.90, 13.21)	− 5.28(− 14.38, 4.75)	C	− 2.44(− 12.88, 8.38)
2.97 (− 4.02, 11.85)	− 2.93(− 11.61, 6.41)	2.44 (− 8.38, 12.88)	D
KS function score improvement			
A	0.12 (− 5.44, 5.91)	2.63 (− 3.11, 8.60)	1.12 (− 4.97, 7.41)
− 0.12 (− 5.91, 5.44)	B	2.31 (− 1.69, 6.24)	0.77 (− 3.53, 5.88)
− 2.63 (− 8.60, 3.11)	− 2.31 (− 6.24, 1.69)	C	− 1.74 (− 6.40, 4.02)
− 1.12 (− 7.41, 4.97)	− 0.77 (− 5.88, 3.53)	1.74 (− 4.02, 6.40)	D
KS knee score improvement			
A	0.05 (− 9.67, 9.38)	2.90 (− 6.95, 12.74)	0.82 (− 9.21, 10.63)
− 0.05 (− 9.38, 9.67)	B	2.85 (− 4.23, 10.00)	0.78 (− 6.74, 8.40)
− 2.90 (− 12.74, 6.95)	− 2.85 (− 10.00, 4.23)	C	− 2.12 (− 10.72, 6.50)
− 0.82 (− 10.63, 9.21)	− 0.78 (− 8.40, 6.74)	2.12 (− 6.50, 10.72)	D

*WMD* weighted mean difference, *95*% *CI* 95% confidence intervals, *KS* knee society. A, The change of PCO decrease of 2 mm or more. B, The change of PCO decrease by 0 mm to 2 mm. C, The change of PCO increase by 0 mm to 2 mm. D, The change of PCO increase by 2 mm or more

In addition, the results (Table 4) of SUCRA of various interventions on the knee joint function post TKR showed that patients with 0 mm ≤ PCO < 2 mm after TKR had a better recovery of joint function than those with the remaining PCO changes (flexion contracture: 28.67%; KS functional score: 78.67%; KS knee score: 75.00%). However, patients with − 2 mm < PCO < 0 mm after TKR presented with a worse recovery of joint function compared with the remaining PCO changes (knee flexion: 14.67%; KS functional score: 31.00%; KS knee score: 36.33%). Moreover, the knee flexion (77.00%) of patients with PCO ≤ − 2 mm after TKR was superior to the other three PCO changes, and the flexion contracture (73.67%) was higher than the other three PCO changes.

**Table 4:** SUCRA of PCO changes on knee joint functions post TKR

	Flexion contracture	Knee flexion	KS function score	KS knee score
A (PCO ≤ − 2 mm)	1 (73.67%)	1 (77.00%)	3 (34.00%)	3 (39.67%)
B (− 2 mm < PCO < 0 mm)	2 (56.33%)	4 (14.67%)	4 (31.00%)	4 (36.33%)
C (0 mm ≤ PCO < 2 mm)	4 (28.67%)	2 (68.00%)	1 (78.67%)	1 (75.00%)
D (PCO ≥ 2 mm)	3 (40.33%)	3 (40.33%)	2 (55.00%)	2 (49.00%)

*KS* knee society. A, The change of PCO decrease of 2 mm or more. B, The change of PCO decrease by 0 mm to 2 mm. C, The change of PCO increase by 0 mm to 2 mm. D, The change of PCO increase by 2 mm or more

### Retrospective analysis of clinical research identifies a better recovery of joint function in patients with 0 mm ≤ PCO < 2 mm after TKR

As illustrated in Table 5, there was no significant difference in the age, gender, BMI, and each functional knee score index of patients with OA before operation (all *p* > 0.05). The changes of PCO were significantly correlated with the flexion contracture, further flexion and KS functional score of patients after TKR (all *p* < 0.05), but showed no correlation with WOMAC and KS knee score (all *p* > 0.05). Among them, each functional knee score of patients with 0 mm ≤ PCO < 2 mm was better than the other three PCO changes (Table 6). Furthermore, changes of PCO were closely related to flexion contracture, further flexion, KS functional score, and KS knee score of patients following TKR (all *p* < 0.05), but not to WOMAC score (*p* > 0.05) (Table 7).

**Table 5:** Comparison of the baseline characteristics of patients with four PCO changes before TKR

	PCO ≤ − 2 mm (*n* = 25)	− 2 mm < PCO < 0 mm (*n* = 43)	0 mm ≤ PCO < 2 mm (*n* = 27)	PCO ≥ 2 mm (*n* = 8)	*p* value
Age (years)	66.20 ± 5.68	64.67 ± 5.61	64.00 ± 6.64	66.50 ± 5.58	0.487
Gender (male/female)	8/17	16/27	12/15	3/5	0.833
BMI (kg/m^2^)	25.95 ± 3.38	28.09 ± 3.28	27.08 ± 3.27	27.05 ± 2.73	0.082
Flexion contracture (°)	6.34 ± 1.04	6.02 ± 0.54	6.28 ± 0.96	5.97 ± 0.84	0.335
Further flexion (°)	120.42 ± 1.95	120.73 ± 2.06	119.94 ± 1.85	118.99 ± 1.78	0.091
WOMAC score	110.56 ± 8.38	108.30 ± 6.41	112.81 ± 9.45	106.50 ± 5.81	0.069
KS knee score	39.36 ± 5.13	40.21 ± 3.59	38.48 ± 2.56	41.00 ± 6.26	0.258
KS function score	49.52 ± 2.71	48.67 ± 3.24	50.37 ± 4.12	50.50 ± 4.72	0.200

The measurement data (mean ± standard deviation) were analyzed using one-way ANOVA and enumeration data were analyzed by chi-square test

**Table 6:** Comparison of functional knee score indexes of patients with four PCO changes 1 year after TKR

	PCO ≤ − 2 mm (*n* = 25)	− 2 mm < PCO < 0 mm (*n* = 43)	0 mm ≤ PCO < 2 mm (*n* = 27)	PCO ≥ 2 mm (*n* = 8)	*p* value
Flexion contracture (°)	0.97 ± 0.46	0.95 ± 0.42	0.57 ± 0.28	0.77 ± 0.23	< 0.001*
Further flexion (°)	120.42 ± 3.11	125.41 ± 2.93	126.87 ± 3.93	123.25 ± 1.46	< 0.001*
WOMAC score	28.76 ± 5.17	28.86 ± 5.79	29.30 ± 5.04	25.00 ± 7.82	0.291
KS knee score	83.24 ± 7.22	82.21 ± 7.24	85.89 ± 3.85	85.38 ± 9.18	0.137
KS function score	70.32 ± 9.29	73.58 ± 6.46	77.26 ± 3.37	75.75 ± 2.87	0.002*

The measurement data (mean ± standard deviation) were analyzed using one-way ANOVA. *p < 0.05 indicated significant difference in the four PCO changes

**Table 7:** Comparison of functional knee score indexes of patients with four PCO changes 1 year before and after TKR

	PCO ≤ − 2 mm (*n* = 25)		− 2 mm < PCO < 0 mm (*n* = 43)		0 mm ≤ PCO < 2 mm (*n* = 27)		PCO ≥ 2 mm (*n* = 8)		*p* value
Flexion contracture (°)	− 5.37	1.12	− 5.07	0.64	− 5.71	0.95	− 5.20	0.71	0.030*
Further flexion (°)	3.81	2.47	4.68	2.31	6.93	3.80	4.26	1.84	< 0.001
WOMAC score	− 81.80	8.77	− 79.44	8.26	− 83.52	9.83	− 81.50	8.07	0.300
KS knee score	43.88	7.19	42.00	7.83	47.41	4.35	44.38	11.73	0.032*
KS function score	20.80	10.01	24.91	7.12	26.89	5.82	25.25	6.84	0.038*

The measurement data (mean ± standard deviation) were analyzed using one-way ANOVA. *p < 0.05 indicated significant difference in the four PCO changes

## Discussion

TKR is an effective and durable treatment method for knee OA and ability to efficiently and accurately predict future risk of TKR in earlier stages of OA has potential important applications; however, a subset of patients experience incomplete pain relief and ongoing dysfunction [6, 26]. Further, PCO has previously been shown to be associated with the improved postoperative functional outcomes following TKR after revision total knee arthroplasty [27]. This study conducted a network meta-analysis and a sequential retrospective cohort study to compare the effect of 4 PCO magnitudes (PCO ≤ − 2 mm, − 2 mm < PCO < 0 mm, 0 mm ≤ PCO < 2 mm, and PCO ≥ 2 mm) on the knee joint function of OA patients following TKR. The experimental results demonstrated that patients with 0 mm ≤ PCO < 2 mm had a relatively better postoperative outcome in the knee joint function when compared with others.

The direct evidence of pairwise meta-analysis showed that patients with 0 mm ≤ PCO < 2 mm had a better knee flexion improvement (WMD = 6.00, 95% CI = 2.49–9.50) following TKR. The magnitude of postoperative PCO has been extensively reported to have a close correlation to the improvement in maximum flexion angle in cruciate-retaining knees after TKR [13]. Femoral PCO increase by 2 mm may have the potential to increase flexion by a mean of 14° relative to the neutral position [28]. However, decreased PCO magnitude has been documented to contribute to instability in flexion [29]. Another study shows that 3-mm PCO decrease may reduce knee flexion by 10° prior to the event of tibiofemoral impingement [9]. Similarly, patients with − 2 mm < PCO < 0 mm after TKR presented with a worse recovery of joint function compared with the remaining PCO changes in the current study. Moreover, PCO of more than 2 mm results in a midflexion instability in OA patients treated with single-radius TKR [16]. In addition, the results (Table 4) of SUCRA clarified that patients in the 0 mm ≤ PCO < 2 mm group after TKR had a better functional recovery than the remaining PCO changes. Therefore, the change of PCO between 0 mm and 2 mm may be better for the postoperative knee flexion improvement.

The network meta-analysis further uncovered that there was no significant difference in the improvement values of flexion contracture, knee flexion, KS functional score, and KS knee score of patients following TKR in response to any changes of PCO (Fig. 3, Table 3). In line with our results, no statistical differences are observed in the flexion contracture, further flexion, Hospital for Special Surgery (HSS) score, KS knee score, or KS functional score between each group (<− 2 mm, − 2–0 mm, 0 to + 2 mm, and > 2 mm) following TKR [20]. In addition, a recent study has showed that either cartilage-based or radiographic PCO changes fail to significantly affect postoperative knee flexion after posterior-stabilized TKR [30]. Furthermore, there emerged no significant differences in the postoperative range of motion or patient-reported outcome measures in patients with PCO increase by more than 3 mm, within 3 mm, and decrease by more than 3 mm [23]. Therefore, all above researches further confirmed our findings.

Subsequently, the results from the retrospective study also revealed a significant correlation between PCO changes and the flexion contracture, further flexion, and KS functional score of patients after TKR, in which each functional knee score of patients with 0 mm ≤ PCO < 2 mm was better than the others. In a previously performed study, femoral PCO of 4.7 times greater than healthy knees after TKR may amplify the risk of knee joint flexion contracture [14]. In addition, a recent study based on 107 patients with end-stage knee OA undergoing primary TKR has demonstrated that the PCO change ≥ 0 mm group (130.40° ± 11.63°) shows greater flexion than the PCO change < 0 mm group (123.80° ± 13.12°) during active weight-bearing one year after TKR [31].

## Conclusion

We combined a network meta-analysis with a clinical retrospective cohort study to compare the effects of four changes of femoral PCO before and after TKR on the knee joint function, suggesting a certain clinical significance for TKA. This study firstly clarified the close relationship between knee joint function and postoperative PCO changes in TKR patients, and found that the optimal PCO change range for patients was 0 mm ≤ PCO < 2 mm, which has certain referential significance for clinical related surgery and postoperative evaluation. Nonetheless, due to the difference in the sample size of the four interventions and the number of studies included in the direct pairwise comparisons between the various interventions, our results may be affected to some degree. More comprehensive studies are therefore needed to give evidence that 0 mm ≤ PCO < 2 mm exerts better clinical efficacy in the postoperative function after TKR.

## Supplementary Information

**Additional file 1 Figure S1**. Flowchart of the literature retrieval and screening results.

**Additional file 2 Figure S2**. NOS diagram for literature quality assessment. NOS=Newcastle-Ottawa Scale.

**Additional file 3 Figure S3**. The standard meta-analysis checklist.

## Data Availability

All data generated or analyzed during this study are included in this published article. The datasets used and/or analyzed during the current study are also available from the corresponding author on reasonable request.

## Abbreviations

PCO: Posterior condyle offset
TKR: Total knee replacement
WMD: Weighted mean difference
SUCRA: Surface under the cumulative ranking curves
OA: Osteoarthritis
NOS: Newcastle-Ottawa Scale
WOMAC: Western Ontario and McMaster Universities
AKS: American Knee Society
CrIs: Credible intervals
ANOVA: Analysis of variance
HSS: Hospital for Special Surgery
